# Assessing robustness of carotid artery CT angiography radiomics in the identification of culprit lesions in cerebrovascular events

**DOI:** 10.1038/s41598-021-82760-w

**Published:** 2021-02-10

**Authors:** Elizabeth P. V. Le, Leonardo Rundo, Jason M. Tarkin, Nicholas R. Evans, Mohammed M. Chowdhury, Patrick A. Coughlin, Holly Pavey, Chris Wall, Fulvio Zaccagna, Ferdia A. Gallagher, Yuan Huang, Rouchelle Sriranjan, Anthony Le, Jonathan R. Weir-McCall, Michael Roberts, Fiona J. Gilbert, Elizabeth A. Warburton, Carola-Bibiane Schönlieb, Evis Sala, James H. F. Rudd

**Affiliations:** 1 Department of Medicine, University of Cambridge, Cambridge, UK; 2 Department of Radiology, University of Cambridge, Cambridge, UK; 3 Cancer Research UK Cambridge Centre, University of Cambridge, Cambridge, UK; 4 Department of Clinical Neurosciences, University of Cambridge, Cambridge, UK; 5 Division of Vascular Surgery, Department of Surgery, University of Cambridge, Cambridge, UK; 6 Division of Experimental Medicine and Immunotherapeutics, University of Cambridge, Cambridge, UK; 7 Department of Medical Imaging, University of Toronto, Toronto, ON Canada; 8 EPSRC Centre for Mathematical Imaging in Healthcare, University of Cambridge, Cambridge, UK; 9 School of Medicine, University of Leeds, Leeds, UK; 10 Oncology R&D, AstraZeneca, Cambridge, UK; 11 Department of Applied Mathematics and Theoretical Physics, University of Cambridge, Cambridge, UK

**Keywords:** Medical research, Medical imaging, Atherosclerosis, Carotid artery disease

## Abstract

Radiomics, quantitative feature extraction from radiological images, can improve disease diagnosis and prognostication. However, radiomic features are susceptible to image acquisition and segmentation variability. Ideally, only features robust to these variations would be incorporated into predictive models, for good generalisability. We extracted 93 radiomic features from carotid artery computed tomography angiograms of 41 patients with cerebrovascular events. We tested feature robustness to region-of-interest perturbations, image pre-processing settings and quantisation methods using both single- and multi-slice approaches. We assessed the ability of the most robust features to identify culprit and non-culprit arteries using several machine learning algorithms and report the average area under the curve (AUC) from five-fold cross validation. Multi-slice features were superior to single for producing robust radiomic features (67 vs. 61). The optimal image quantisation method used bin widths of 25 or 30. Incorporating our top 10 non-redundant robust radiomics features into ElasticNet achieved an AUC of 0.73 and accuracy of 69% (compared to carotid calcification alone [AUC: 0.44, accuracy: 46%]). Our results provide key information for introducing carotid CT radiomics into clinical practice. If validated prospectively, our robust carotid radiomic set could improve stroke prediction and target therapies to those at highest risk.

## Introduction

Carotid CT angiography (CTA) is commonly performed following an ischaemic stroke or transient ischaemic attack (TIA) to help guide patient management, for example between carotid endarterectomy surgery plus medical therapy or medical therapy alone. Carotid CTA imaging allows measurement of carotid artery luminal stenosis and unenhanced images provide information about calcification of the artery wall. The decision to perform carotid endarterectomy surgery, to reduce future stroke risk, is based on the degree of carotid stenosis and the presence of relevant symptoms. However, whilst stenosis provides important information about disease burden, it does not inform about the underlying plaque stability or degree of inflammation and patients may have second events with only mild to moderate carotid artery narrowings^1^.

Radiomics, sometimes called ‘texture analysis’, comprises image analysis methods that involve the high-throughput extraction of minable imaging features^2^ from radiological images. Radiomic features quantify simple and complex patterns in the data, such as the roundness of a tumour, the spatial arrangement of voxels or variations in signal intensity across a lesion of interest. These features have been used to develop diagnostic and prognostic prediction models, particularly in oncology^3,4^ where radiomic features can predict lung and other cancer survival times better than the gold standard TNM (Tumour, Node, Metastasis) staging system^5–7^. There is growing interest in the application of radiomics to cardiovascular imaging^8–11^, for example to differentiate causes of prosthetic valve obstruction using cardiac computed tomography (CT) radiomic features^9^, to distinguish between hypertensive and hypertrophic cardiomyopathy from magnetic resonance imaging (MRI)^10^, or to characterise carotid artery plaques from ultrasound images^12,13^.

A radiomic biomarker should be reproducible, robust and accurate^14^. However, radiomic features are susceptible to variations^15,16^, including image acquisition (e.g. use of different CT scanner manufacturers and models, acquisition protocols and image reconstruction methods), image segmentation (e.g. inter-observer and intra-observer variability in delineating the region-of-interest [ROI]/volume-of-interest [VOI]) and at the feature extraction stage (e.g. use of different radiomics software, different image pre-processing settings or radiomic feature definitions). To minimise such variations^6^, there is a growing call for the standardisation of protocols at every stage of the radiomics workflow^17^. Where harmonisation is not possible (e.g. using the same type of CT scanner in every hospital), robustness analyses are essential in determining the extent to which such variations can be tolerated for each specific application, i.e. without affecting predictive performance. Robustness analyses evaluate the impact of changes in these parameters on radiomic features, aiming to find those most immune to such perturbations. These ‘robust’ features are expected to perform well when tested on new image datasets, a characteristic referred to as ‘good generalisability’^18^.

The majority of published robustness and repeatability studies have been conducted using phantoms^19,20^ and restricted to oncology^21–23^, such as in non-small cell lung cancer^16^ or oesophageal cancer^24^. In cardiovascular imaging, there have been relatively few studies—one using a phantom in single photon emission computed tomography^25^ and the other finding robust myocardial radiomic features from cardiac MRI^26^. However, feature robustness is specific to the disease phenotype being studied and to the imaging modality used^18^. Therefore, there is an unmet need for the assessment of radiomic robustness in cardiovascular disease, specifically in carotid CT angiography (CTA) imaging.

In this study, we first investigated the robustness of 93 individual carotid CTA radiomic features following ROI/VOI perturbations under different CTA image pre-processing and across single vs multiple artery slice situations. We then determined (1) the optimal image pre-processing settings (i.e. the settings that provided the highest proportion of radiomic features with excellent robustness) and (2) the most robust and non-redundant (i.e. not highly correlated) radiomic features for machine learning classification of culprit *versus* non-culprit carotid arteries in patients with prior cerebrovascular events.

In summary, we sought to understand whether radiomic features extracted from standard clinical CT scans were robust and reliable and whether they could provide additional prognostic information to help identify higher-risk culprit arteries from lower-risk non-culprit carotid arteries.

## Results

Carotid CTA scans from 41 patients with previous stroke or TIA were analysed in this study comprising 41 culprit and 41 non-culprit carotid arteries (82 carotid arteries in total). The clinical characteristics of the patients and the plaque characteristics of their carotid arteries are shown in Table 1.

**Table 1:** Patient and culprit versus non-culprit carotid characteristics.

Patient characteristics (n = 41)			
Age (years)—mean (SD)	74.1 (8.4)		
Male—n (%)	32 (78.0%)		
*Presence of cardiovascular risk factors*			
Diabetes mellitus—n (%)	8 (19.5%)		
Hypertension—n (%)	27 (65.9%)		
Smoking history (current or former)—n (%)	29 (70.7%)		
*Cerebrovascular event*			
Stroke—n (%)	30 (73.2%)		
TIA—n (%)	11 (26.8%)		

Carotid characteristic	Culprit (n = 41)	Non-culprit (n = 41)	*p* value
*Plaque type*			
Calcified—n (%)	9 (22.0%)	15 (36.6%)	0.070
Noncalcified—n (%)	2 (4.9%)	5 (12.2%)	0.250
Mixed—n (%)	30 (73.2%)	21 (51.2%)	0.012*
*Calcium burden (AU)*			
Median (IQR)	263 (95–701)	387 (63–659)	0.706
Minimum	0	0	-
Maximum	1963	1671	-
*Carotid stenosis (%)*			
Mean (SD)	72 (17)	40 (22)	4.70 × 10^–9^***
Minimum	29	3	-
Maximum	99	88	-

Plaque type comparisons: McNemar’s test on paired proportions; Calcium score comparison: Wilcoxon signed-rank test; Carotid stenosis comparison: paired Student t-test. n, number of carotids or number of patients respectively; AU, Agatston units; IQR, interquartile range, SD, standard deviation; TIA, transient ischaemic attack; * *p* value < 0.05, *** *p* value < 0.001.

## Assessing the ability of morphological operations to capture inter-observer segmentation variability

**Figure 1:**
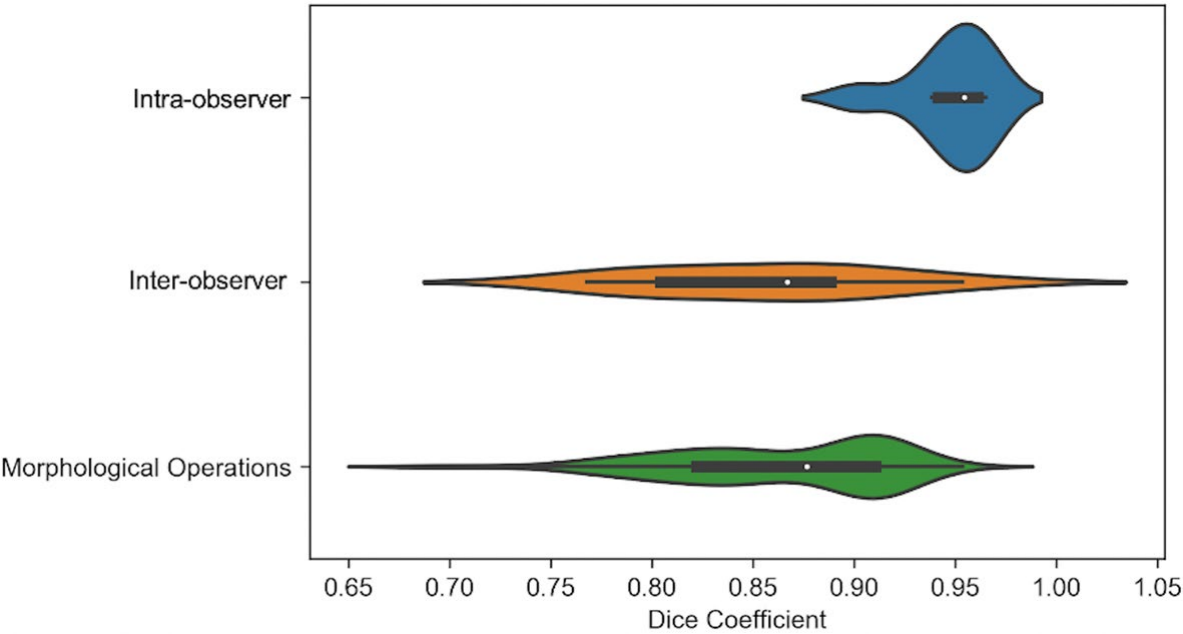
Violin plots of image segmentation agreement as determined by the Dice coefficient with the original ROI. Intra-observer variability was determined by manual segmentation of the ROIs by the same observer (EPVL) at two separate time points for 8 carotid arteries. Inter-observer variability was determined by manual ROI segmentation by two independent observers (EPVL and CW), performed on 8 carotid arteries. The ROIs drawn by the primary observer (EPVL) on 82 carotid arteries were compared with those generated following dilation and erosion morphological operations in single-slice analysis to determine the Dice coefficient distribution of systematic ROI perturbations. The violin plots each contain a black box-plot of the Dice coefficients, with the white dot representing the median Dice coefficient. Whereas the box-plots correspond to the actual data points of the Dice coefficients, the coloured shapes provide a visualisation of the underlying Dice coefficient distributions via kernel density estimations.

We found that there was low variability in intra-observer segmentation, as shown in Fig. 1, but there was greater variability in inter-observer segmentation. The morphological operations applied to the ROIs captured the range of variability that occurred with human inter-observer variability, demonstrated in Fig. 1.

## Assessing feature robustness in different image configurations

### Feature robustness in single-slice analysis

Over 50% of radiomic features that were extracted had excellent robustness to ROI perturbations when using the original image (i.e. no prior normalisation or resegmentation) and a fixed BW for image quantisation, ranging from 10 to 35 (in increments of 5). Using a fixed BW of 10 led to a higher proportion of poorly robust (ICC < 0.5) radiomic features compared with the other BWs, see Supplementary Fig. S1.

The best BW setting (i.e. had the most features with excellent robustness) for single-slice analysis with no image pre-processing was BW 25–30. This corresponds with the PyRadiomics default setting (fixed BW of 25) and a detailed breakdown of the radiomic features by robustness category and feature class type for this setting is provided in Supplementary Table S1. Overall, using a fixed BW of 25 in single-slice analysis resulted in 52.7% of radiomics features having excellent robustness, 35.5% having moderate robustness and 11.8% having poor robustness. Using a fixed BW rather than a fixed BN (from 8 to 256) for image quantisation led to a higher proportion of radiomic features with excellent robustness. If using fixed BNs as the method for image quantisation, the upper limit of BNs investigated (from 8 to 256 in powers of 2) led to the highest proportion of poorly robust radiomic features (15.1% of radiomic features had poor robustness, whilst the proportions were < 10% for other BNs).

Prior normalisation of the image reduced the proportion of poorly robust radiomic features but did not impact the proportion of radiomic features with excellent robustness, when compared to no prior normalisation. In the prior normalisation pre-processing setting, only different BNs could be investigated as using different BWs in the range of 10–35 led to ROIs with too few grey values for radiomic feature calculation.

Resegmentation in single-slice analysis reduced the proportion of radiomic features with excellent robustness (from 52.7 to 8.6%), but also reduced the proportion of poorly robust radiomic features as compared to no resegmentation (from 11.8 to 3.2%). In resegmentation, the majority of radiomic features had moderate robustness against ROI perturbations, 88.2% compared with only 35.5% when no image pre-processing was applied.

Across the 19 different image settings investigated (see Supplementary Table S2), only 2 radiomic features (a) GLDM: Large Dependence High Grey Level Emphasis and (b) GLRLM: Long Run High Grey Level Emphasis, demonstrated excellent robustness across all 19 settings (100%). However, 61 out of 93 (65.6%) extracted radiomic features showed excellent robustness in at least 1 setting, see Supplementary Fig. S2.

### Feature robustness in multi-slice analysis

In multi-slice analysis, the image quantisation method leading to the highest proportion of radiomic features with excellent robustness involved using fixed BWs, as opposed to fixed BNs, consistent with our single-slice analysis findings. Over 55% of all radiomic features had excellent robustness when using the original image in multi-slice analysis, with similar proportions across all BW settings from 10 to 35. There was a slight decrease in performance between BW 30 to BW 35 (BW 30; 58.1% excellent robustness, 9.7% poor robustness; BW 35; 55.9% excellent robustness, 11.8% poor robustness).

Similar to our single-slice analysis findings, prior normalisation in multi-slice analysis reduced the proportion of poorly robust features but had little impact on the proportion of excellent robustness radiomic features. Following resegmentation, the proportion of features with excellent robustness decreased (from 55.9% to 15.1%), however, so did the proportion of poorly robust features (from 10.8% to 2.2%). The majority of radiomic features shifted to moderate robustness, 82.8% compared with 33.3% when no image pre-processing was applied. Table 2 provides a breakdown of the different radiomic feature classes by robustness category (excellent, moderate and poor) in multi-slice analysis following (A) no image pre-processing and (B) resegmentation.

**Table 2:** Number of radiomic features with excellent, moderate and poor robustness by feature class in multi-slice analysis.

Feature class	Excellent robustness ICC ≥ 0.9	Moderate robustness 0.5 ≤ ICC < 0.9	Poor robustness ICC < 0.5
Multi-slice Analysis: Original, fixed bin width = 25, B-spline interpolation			
First order (n = 18)	10 (55.6%)	6 (33.3%)	2 (11.1%)
GLCM (n = 24)	17 (70.8%)	7 (29.2%)	0 (0.0%)
GLDM (n = 14)	5 (35.7%)	7 (50.0%)	2 (14.3%)
GLRLM (n = 16)	11 (68.8%)	1 (6.3%)	4 (25.0%)
GLSZM (n = 16)	6 (37.5%)	8 (50.0%)	2 (12.5%)
NGTDM (n = 5)	3 (60.0%)	2 (40.0%)	0 (0.0%)
Total (n = 93)	52 (55.9%)	31 (33.3%)	10 (10.8%)
Multi-slice Analysis: Resegmentation, fixed bin width = 25, B-spline interpolation			
First order (n = 18)	3 (16.7%)	13 (72.2%)	2 (11.1%)
GLCM (n = 24)	2 (8.3%)	22 (91.7%)	0 (0.0%)
GLDM (n = 14)	3 (21.4%)	11 (78.6%)	0 (0.0%)
GLRLM (n = 16)	3 (18.8%)	13 (81.3%)	0 (0.0%)
GLSZM (n = 16)	3 (18.8%)	13 (81.3%)	0 (0.0%)
NGTDM (n = 5)	0 (0.0%)	5 (100.0%)	0 (0.0%)
Total (n = 93)	14 (15.1%)	77 (82.8%)	2 (2.2%)

Definition of ICC used = 2-way mixed-effects model, absolute agreement, single rater intraclass correlation coefficient; n, number of radiomics features.

Across the 19 image settings investigated in multi-slice analysis (see Supplementary Table S2), 4 radiomic features demonstrated excellent robustness across all settings (100%), these were: a) GLDM: Grey Level Variance, b) First Order: Mean Absolute Deviation, c) GLRLM: Grey Level Variance and d) GLDM: Large Dependence High Grey Level Emphasis. In at least 1 setting, 67 out of 93 (72%) extracted radiomic features showed excellent robustness, see Supplementary Fig. S3.

### Radiomic feature robustness similarities and differences in single-slice and multi-slice approaches

**Figure 2:**
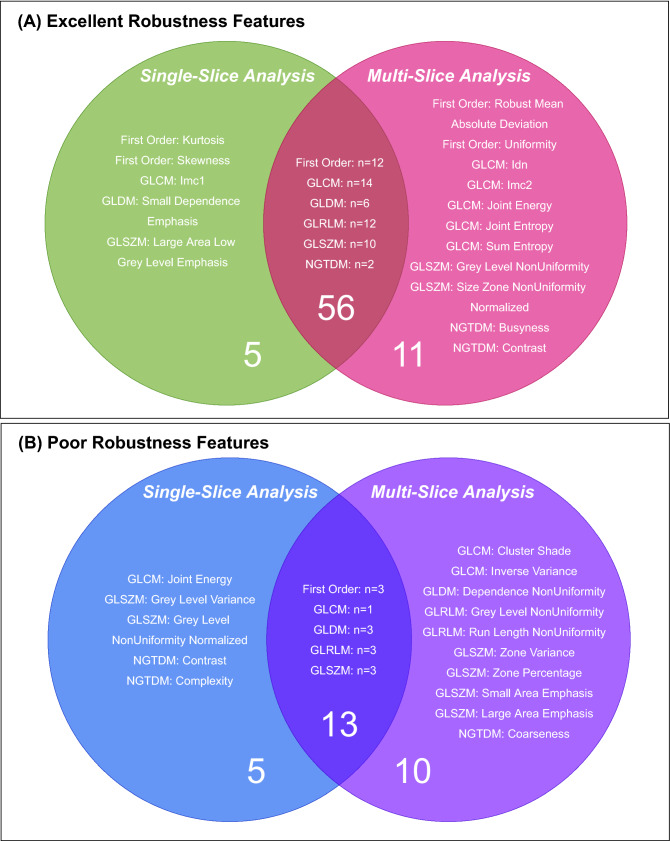
Venn diagram to show the radiomic features that had (**A**) Excellent robustness in single-slice and/or multi-slice analysis and, (**B**) Poor robustness in single-slice and/or multi-slice analysis.

In single-slice analysis, 61 features had excellent robustness in at least one image setting out of the 19 settings investigated, whilst 67 features had excellent robustness in multi-slice analysis. There was considerable overlap in the radiomic features with excellent robustness between single-slice and multi-slice approaches (n = 56), these included First Order: Variance and GLCM: Autocorrelation. However, there were also radiomic features that had excellent robustness in the single-slice approach only (n = 5) such as First Order: Kurtosis and GLDM: Small Dependence Emphasis or the multi-slice approach only (n = 11) such as First Order: Uniformity and GLCM: Joint Energy, these are illustrated in Fig. 2A.

In single-slice analysis, 18 features were identified as poorly robust in at least 1 out of the 19 settings investigated, whilst 23 features were identified as poorly robust in multi-slice analysis. There were no radiomic features that were poorly robust in all 19 settings. Figure 2B illustrates the radiomic features that were identified as poorly robust in both single-slice and multi-slice analysis (n = 13) such as First Order: 10th Percentile and GLDM: Low Grey Level Emphasis, as well as those unique to single-slice (n = 5) analysis such as GLSZM: Grey Level Variance and NGTDM: Contrast or multi-slice analysis (n = 10) such as GLCM: Inverse Variance and NGTDM: Coarseness.

## Multi-slice analysis: Impact of PyRadiomics interpolation method

The proportion of robust radiomic features was similar whether B-spline or linear interpolation was used to resample the 3 mm slice thickness images and VOI segmentation masks to 1 × 1 × 1mm^3^. The vast majority of radiomic features showed excellent absolute agreement and consistency between the two methods of interpolation. No features were poorly robust with regards to the method of interpolation used. The breakdown by radiomic feature class is shown in Supplementary Table S3.

## Culprit *versus* non-culprit carotid arteries: Machine learning classification

Since a fixed bin width of 25 was found to produce the highest proportion of radiomic features with excellent robustness, we used the radiomic features extracted using this image quantisation level in the following image settings for machine learning classification: (1) single-slice approach: original image, (2) single-slice approach: with resegmentation, (3) multi-slice approach: original image and (4) multi-slice approach: with resegmentation.

### Non-redundant radiomic feature sets with excellent robustness in single-slice and multi-slice approaches

Different sets of non-redundant radiomic features with excellent robustness were identified depending on the image setting used (1–4), these radiomic features are detailed in Supplementary Table S4. For single-slice analysis using the original image, this consisted of 14 radiomic features; following resegmentation, this comprised 7 radiomic features. For multi-slice analysis using the original image, this consisted of 14 radiomic features that decreased to 10 radiomic features following resegmentation.

### Machine learning classification performance determined by five-fold cross-validation

Several machine learning classifiers were investigated in a five-fold cross-validation scheme using (1) carotid calcium score as the only predictor, (2) radiomic features (non-redundant with excellent robustness) as the only predictors and (3) radiomic features with carotid calcium score (termed the ‘integrated model’) as predictors to differentiate culprit from non-culprit carotid arteries. The image setting that led to the highest predictive performance was the multi-slice approach with resegmentation (image setting 4). Within this setting, the best performing machine learning classifier amongst those investigated was the Elastic Net logistic regression-based classifier. Elastic Net regression uses a mixture between *L*_1_ and *L*_2_ regularisation whereby *L*_1_ regularisation reduces the coefficients of certain features to zero, thereby reducing the number of variables in a model (i.e. sparse feature selection) and the *L*_2_ penalty term constrains the magnitude of the feature coefficients so that a model is not dominated by any single feature. In this image setting and using this best performing model (Elastic Net, weight for *L*_1_ and *L*_2_ penalties = 0.5), carotid calcium score alone was a poor predictor of culprit *versus* non-culprit carotid artery status, see Supplementary Table S5.

**Figure 3:**
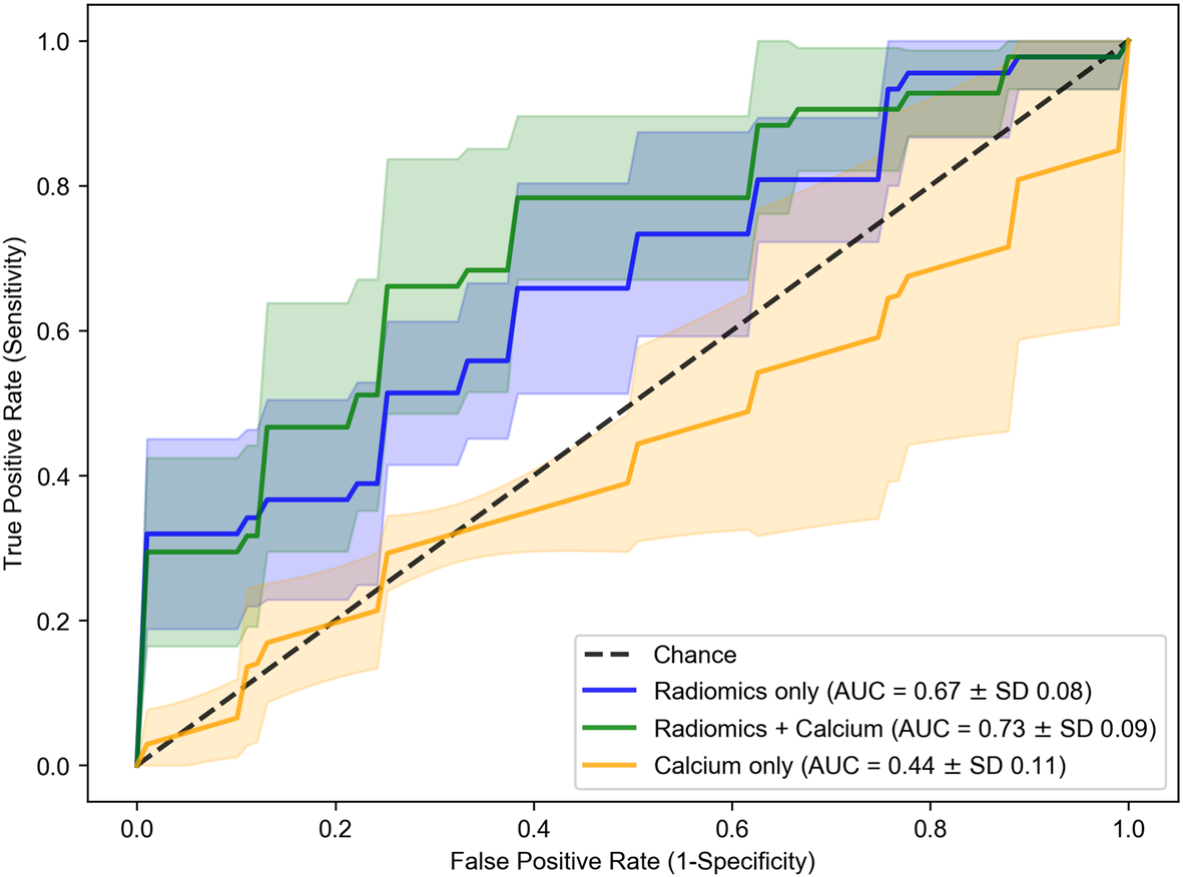
Mean receiver operating characteristic (ROC) curves of five-fold stratified cross-validation in multi-slice analysis with resegmentation for Elastic Net logistic regression (weight for L_1_ and L_2_ penalties = 0.5) using (1) radiomic features only as predictors, (2) radiomics features and calcium and (3) calcium only. Image setting: multi-slice analysis with resegmentation and a fixed bin width of 25. Dashed line indicates expected AUC for a random chance classifier. AUC, area under the ROC curve; SD, standard deviation.

The mean (standard deviation, SD) area under the receiver operating characteristic curve (AUC) for carotid calcium score alone was 0.44 (0.11) and the mean (95% confidence intervals [CI]) accuracy was 46% (25–56%), see Fig. 3. Please note, in five-fold cross-validation, an AUC is provided for the model performance in each fold. The mean cross-validated AUC is the average of the AUC values across the five folds.

Using radiomic features (with resegmentation to [0, 200 HU]) alone as predictors performed better than carotid calcium alone, with a mean (SD) AUC of 0.67 (0.08) and a *p*-value of 0.043. The combination of radiomic features with carotid calcium as predictors led to the highest predictive performance, with a mean (SD) AUC of 0.73 (0.09), a mean (95% CI) accuracy of 69% (47–88%) and a *p*-value of 0.043 when compared with carotid calcium alone and a *p*-value of 0.042 when compared with radiomic features alone. The performance (mean AUC with SD) of the other machine learning classifiers using radiomic features and carotid calcium as predictors were: decision tree 0.58 (0.19), random forest 0.67 (0.08), LASSO 0.72 (0.09), neural network 0.60 (0.09) and XGBoost 0.56 (0.09). Please see Supplementary Table S6 for the sensitivity and specificity of each individual model.

**Figure 4:**
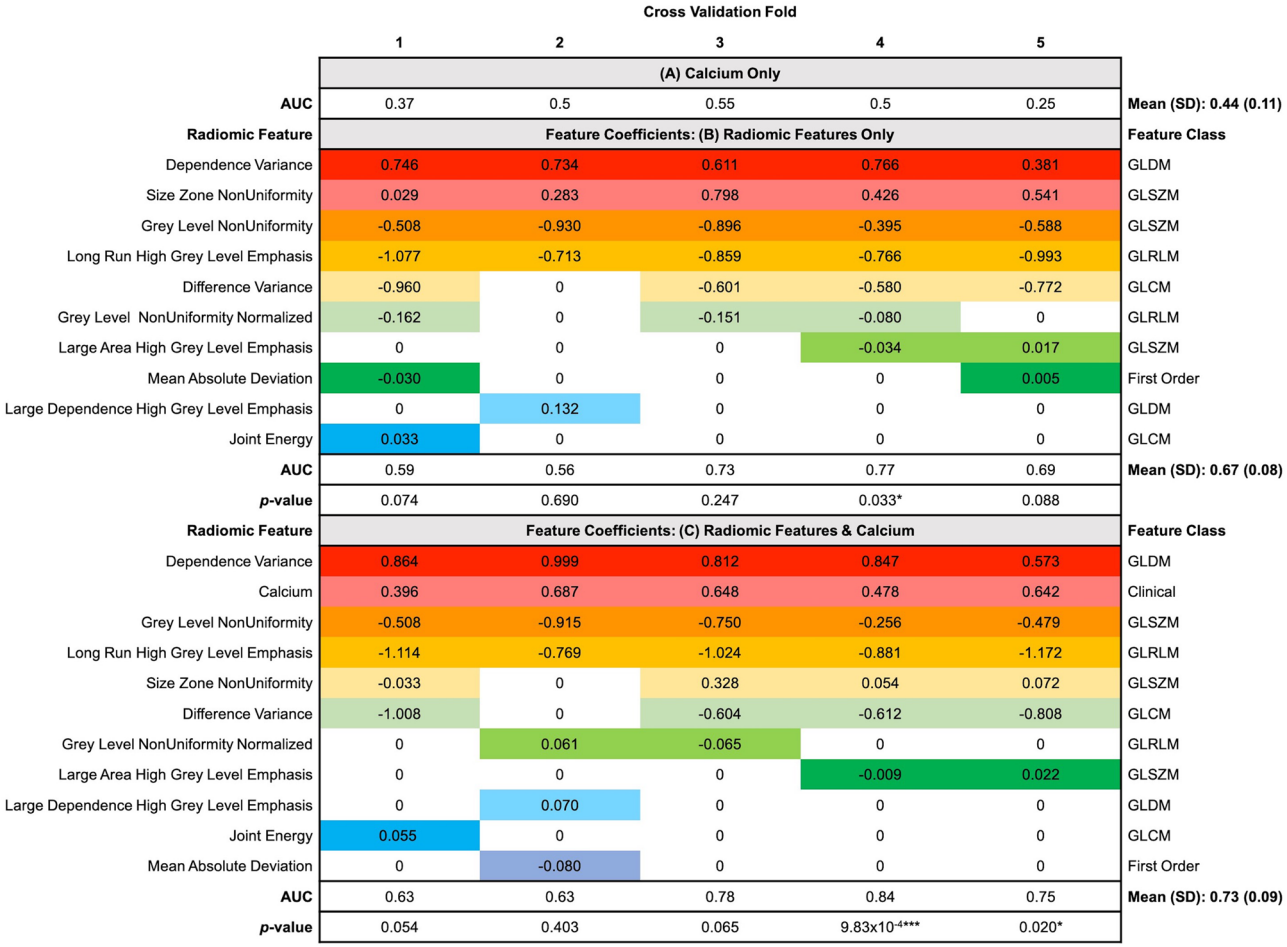
AUC and feature coefficients of predictors used in the Elastic Net logistic regression classifier in multi-slice analysis with resegmentation. The coefficients of the non-redundant radiomic features with excellent robustness in multi-slice analysis with resegmentation according to the Elastic Net model per fold of the five-fold cross-validation scheme are provided. The name of these radiomic features are provided on the left-hand side, whilst their corresponding feature classes are given on the right-hand side of the table. The AUC for each fold of cross-validation is provided and the mean AUC and standard deviation are stated in bold. The predictive performance of (**A**) when calcium is used as the only predictor, (**B**) when only radiomic features are the predictors, and (**C**) when using both radiomic features and calcium in an integrated model are provided. * p-value < 0.05, *** p-value < 0.001 when comparing the classification performance per fold of radiomic models **B** and **C** with the calcium only model (**A**) using DeLong’s method. The colours highlight non-zero feature coefficients where non-zero coefficients indicate how features played a role in the model’s predictions.

The radiomic feature set (n = 10) for multi-slice analysis with resegmentation is shown in Fig. 4 along with the coefficients for each feature as determined by the Elastic Net classifier per fold within the cross-validation scheme. The feature coefficients indicate the importance of the features for the model’s predictions. Larger positive coefficient values suggest higher importance for predicting the culprit carotid artery class, whilst larger negative coefficient values suggest higher importance for predicting the non-culprit carotid artery class.

Overall, there were 3 radiomic features that were highly consistent in being relevant predictors for carotid artery status across every cross-validation fold: (1) GLDM: Dependence Variance, (2) GLSZM: Grey Level NonUniformity and (3) GLRLM: Long Run High Grey Level Emphasis.

## Discussion

To our knowledge, this is the first systematic approach to evaluate the robustness and reproducibility of carotid CT angiography radiomics and its impact on the ability to identify culprit carotid arteries in stroke and TIA patients. We revealed factors that impacted robustness and identified a radiomics set that could be used to predict patient symptom state. We compared our robust feature set with carotid calcium scoring using several machine learning models, demonstrating superior performance.

The ROI perturbations (morphological operations: dilations and erosions) that we used mimicked the ROI over- and under-estimation variations introduced by human subjectivity in clinical practice (inter-observer variability) when using manual delineation methods. Not all of the 93 radiomic features that we extracted were robust against these morphological perturbations and the proportion of radiomic features with excellent robustness varied depending on the image settings used.

We found that without image pre-processing, the majority of radiomic features (but not all) had excellent robustness against ROI perturbations. There were similarities between the radiomic features with poor robustness in both single-slice and multi-slice analysis to include the radiomic features: First Order: 10th Percentile and GLDM: Low Grey Level Emphasis. These radiomic features are related to low grey values within the CTA image and therefore most likely reflect the varying amounts of carotid artery perivascular fat captured in the segmentation mask following the morphological perturbations. Following grey value range resegmentation, which restricted radiomic feature calculation to Hounsfield units between 0 and 200 inclusive, the proportion of poorly robust radiomic features was greatly reduced as the low grey values that reflect perivascular fat were excluded. However, resegmentation also reduced the number of radiomic features with excellent robustness and shifted them into the moderate robustness category.

Whereas prior image normalisation appears necessary for image pre-processing of MRI scans for radiomic work (where the grey values are arbitrary), it does not seem necessary for carotid CTA scans (where grey values are calibrated to Hounsfield units). In our study, prior normalisation of CTA scans with PyRadiomics did not increase the proportion of radiomic features with excellent robustness. This is in line with most CT imaging radiomic studies that do not tend to apply prior normalisation^27^.

The use of bin number *versus* bin width as image quantisation methods were not interchangeable. The radiomic features in one setting were not necessarily robust in another setting. We found that a fixed bin width of 25 or 30 for image quantisation led to the greatest proportion of radiomic features with excellent robustness. Since the PyRadiomics default is already a fixed bin width of 25, we recommend use of that setting in future carotid CTA radiomics studies. In the bin width settings that were investigated in this study (from 10 to 35 in increments of 5), we found a decrease in the proportion of radiomic features with excellent robustness when using the higher limit of 35, and so we did not investigate higher values than this. When using fixed bin numbers as the method for image quantisation, we found that using BN = 256 led to the highest proportion of poorly robust features.

For the different image settings, we investigated both a single-slice approach and a multi-slice approach. Multi-slice analysis, which involved ROI delineation of the carotid artery on multiple carotid CTA axial slices, produced a higher proportion of radiomic features with excellent robustness than single-slice analysis, which used only one CTA axial slice at the carotid artery bifurcation. Although there was a considerable overlap between the radiomic features with excellent robustness in both single-slice and multi-slice approaches, there were certain radiomic features that had excellent robustness only in either single-slice analysis or multi-slice analysis. This was also the case for radiomic features with poor robustness; certain radiomic features had excellent robustness in multi-slice analysis, but poor robustness in single-slice analysis, for example GLCM: Joint Energy. Single-slice and multi-slice analysis approaches may reveal different radiomic features that are robust because the features extracted from a single-slice are more dependent on ROI placement around the carotid bifurcation, whereas radiomic features extracted during multi-slice analysis include more voxels, capturing more information about the carotid artery. The multi-slice approach led to the highest proportion of robust radiomic features and had better predictive value than the single-slice approach. These results echo those in oncology radiomic studies, which found that using a multi-slice approach compared to a single-slice approach (i.e. whole tumour analysis *versus* the largest cross-sectional area) was more representative of tumour heterogeneity^28^.

For the identification of culprit *versus* non-culprit carotid arteries in symptomatic patients, we investigated several machine learning algorithms which have been extensively applied to radiomics and quantitative imaging^29–31^. This approach acknowledged the “no free lunch” theorem^32^—that there is no universal best model for every task^33^. In our study, the ElasticNet model achieved the highest performance amongst those investigated. We identified 10 non-redundant radiomic features with excellent robustness when using a multi-slice approach with grey value range resegmentation ([0, 200] HU) that significantly outperformed carotid calcium scoring in machine learning classification. As a univariable predictor, carotid calcium had poor predictive performance. This was not surprising since there was no statistically significant difference between the carotid calcium score of culprit and non-culprit carotid arteries. Subsequently, the best predictive model consisted of the radiomic feature set with resegmentation to [0, 200] HU, alongside carotid calcification. This may reflect how resegmentation excluded high grey values, largely related to carotid calcification and luminal contrast, so that differences between culprit and non-culprit carotid artery radiomic profiles could be more easily identified. In addition, this demonstrates that carotid calcium and the information captured by our radiomic features after resegmentation are complementary.

Other groups have also reported using metrics derived from CTA images, for example, Gupta et al*.* investigated the discriminative ability of CTA plaque thickness measurements to identify symptomatic carotid artery stenosis^34^. This indicates that carotid CTA imaging contains information beyond luminal stenosis and in this proof-of-principle study, we have shown that radiomics is a feasible and reliable approach to extract this information.

Overall, our findings suggest that: (1) a multi-slice approach is better than a single-slice approach in terms of radiomic feature robustness and predictive accuracy, (2) there is no need for image normalisation in carotid CTA radiomic studies, (3) grey value range resegmentation can help improve predictive accuracy and (4) because radiomic features can be susceptible to changes in the imaging and radiomics workflow, it is important that future studies include detailed descriptions of the image settings used to ensure reproducibility and replicability. This information would ideally include the image acquisition protocols, image pre-processing details and method of interpolation, method and value of image quantisation, and radiomic feature definitions.

## Limitations

One limitation of this study is its retrospective nature—the carotid imaging datasets were pooled from three prior vascular imaging studies: ICARUSS^35^, VISION^36^ and CHAI^37^. Additionally, all images were acquired using the same scanner in one centre. Consequently, the robust radiomic features identified here may be specific to datasets derived in similar settings. In addition, our imaging dataset captured information from culprit carotid arteries after plaque rupture had occurred. Ideally, we would highlight high risk arteries before that stage. Now that we have identified CT-based carotid radiomic features that are robust, a prospective study of at-risk patients using different hardware manufacturers will be an important next step.

We also acknowledge that the use of a 3 mm slice thickness may result in loss of some information and might lead to partial volume effects. Nevertheless, even using this slice thickness, we did identify a robust subset of features that could classify carotid plaques with reasonable accuracy. Further work should test the hypothesis that thinner image slices perform better.

Another consideration is that we used anatomical criteria to standardise the region of the carotid artery that was segmented for single-slice analysis (i.e. the axial slice through the bifurcation) and multi-slice analysis (14 slices about the carotid bifurcation). A possible limitation of this approach, particularly for single-slice analysis, is that the responsible carotid plaque may not be fully captured in the ROI. To account for this, we tested the predictive performance of a multi-slice analysis. We also investigated the impact of prior resegmentation of the image to limit the HU values analysed and counter possible differences in arterial contrast densities.

Finally, here we investigated only unfiltered radiomic features. There are other radiomic parameters that can be extracted after image filtering, such as Gabor filters and wavelet transformations. However, as this was a first proof-of-principle study using first-order and higher-order radiomic features, we wanted to limit the number of features extracted. Future work could expand on this. As the primary objective of this study was robustness analysis rather than developing a definitive radiomics signature, the default Python scikit-learn configurations for the machine learning classifiers were used, without extensive hyperparameter tuning. This avoided further reduction of the limited dataset that could be used for training the machine learning classifiers. Nevertheless, it may be that the predictive performance we have already achieved could be bettered with hyperparameter tuning in future work.

## Conclusion

In summary, to the best of our knowledge, this is the first systematic approach to evaluate the robustness and reproducibility of CT radiomics in carotid artery atherosclerosis. We identified a set of radiomic features that are robust, non-redundant and have superior predictive performance, over and above the degree of calcification, for the classification of culprit *versus* non-culprit carotid arteries in patients with stroke and TIA. If validated prospectively, this carotid CT radiomic features set could improve stroke prediction and target therapies to those at highest risk.

## Methods

### Carotid CT dataset

This study used carotid CTA scans pooled from three observational vascular imaging research datasets from a single institution (Addenbrooke’s Hospital, Cambridge University Hospitals National Health Service Foundation Trust, Cambridge, UK)^35–37^. All studies had appropriate ethical approvals in place by the Cambridge Central Research Ethics Committee; informed consent was obtained from all patients and the studies were conducted according to relevant guidelines and regulations. The studies had similar inclusion and exclusion criteria, which are listed in the published papers^35–37^. All participants had experienced a carotid artery-related ischaemic stroke or TIA during the 3 months before imaging.

In total, data from 41 patients were included, comprising 82 carotid arteries (41 culprit and 41 non-culprit). The culprit carotid artery was determined by the side consistent with the clinical presentation of stroke (or TIA) symptoms, and the non-culprit carotid artery was defined as the artery contralateral to the culprit. Further details of how the culprit carotid plaque was identified and how carotid images with and without contrast were acquired using a standard clinical protocol are described in Supplementary Methods S1.

### Image analysis

**Figure 5:**
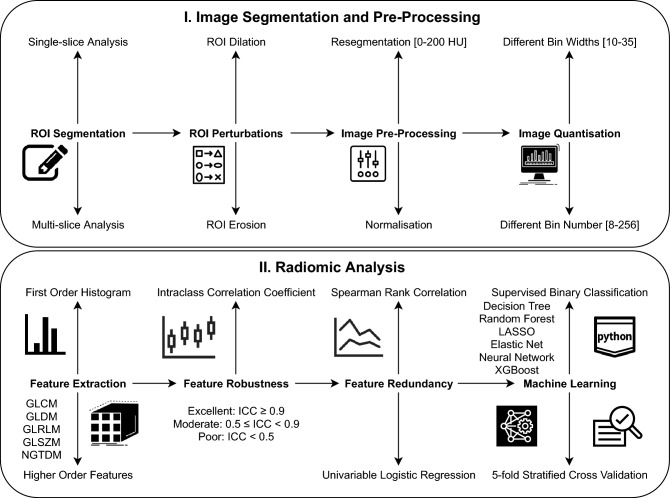
Radiomics workflow. The upper panel illustrates the steps taken from manual segmentation of the carotid CTA images to create ROIs for single-slice analysis (and VOIs for multi-slice analysis) to segmentation mask perturbations, prior image normalisation or resegmentation and image quantisation. The lower panel outlines the subsequent process of radiomic features extraction, robustness analysis and machine learning for the differentiation of culprit versus non-culprit carotid arteries. The predictive ability of the classifiers was assessed via five-fold stratified cross-validation.

Figure 5 illustrates the radiomics workflow within this study. All CT images were analysed by a reader (EPVL) blinded to the clinical status of the carotid artery. Where a second reader is mentioned, (CW), they were also blinded in the same fashion. Details of the methodology used to assess carotid artery plaque characteristics are found in Supplementary Methods S2, and details of intra- and inter-observer reproducibility evaluation are provided in Supplementary Methods S3.

#### Manual segmentation: single-slice analysis

In single-slice analysis, one axial CTA slice, at the carotid bifurcation, was used on each side, with original slice thickness of 0.625 mm and slice spacing of 0.4 mm. ROIs were drawn to encompass the whole vessel as closely as possible, including the outer wall, using commercially available research software (TexRad; Feedback Medical Ltd, Cambridge, UK).

#### Manual segmentation: multi-slice analysis

CTA slices were resampled to 3 mm slice thickness using the OsiriX MD software resampling plugin (Pixmeo SARL, Bernex, Geneva, Switzerland) as per published methods^35–37^. 14 consecutive carotid artery slices were manually segmented using TexRad (as in single-slice analysis) with ROIs drawn around the carotid artery adventitia, with the carotid bifurcation designated as slice zero^35–37^. Reads incorporated all slices from 3 below the carotid bifurcation to 10 slices above, covering portions of the common carotid and internal carotid arteries. For each carotid artery, the 14 consecutive slices were amalgamated into a single VOI from which radiomic features were subsequently extracted.

#### Radiomics feature extraction

PyRadiomics is an open-source Python package developed for the standardisation of radiomic feature extraction^38^. PyRadiomics and Python were used for feature extraction from the ROIs and VOIs described above. Six feature classes were extracted: (1) first-order intensity histogram statistics, (2) Grey Level Co-occurrence Matrix features (GLCM)^39,40^, (3) Grey Level Run Length Matrix features (GLRLM)^41^, (4) Grey Level Size Zone Matrix (GLSZM)^42^, (5) Grey Level Dependence Matrix (GLDM)^43^ and (6) Neighbouring Grey Tone Difference Matrix Features (NGTDM)^44^. Please see Supplementary Table S7 and S8 for details of the individual extracted radiomic features.

### Robustness analysis

#### ROI perturbations

Manual segmentation (as opposed to automatic segmentation) is a source of intra- and inter-observer variability. Automatic segmentation methods are not currently widely available in medicine, although this is an area of active development. We therefore evaluated the impact of perturbations to ROI delineation on the extracted radiomic features by systematically performing ROI dilation and erosion. This was to simulate certain variations in ROI/VOI placement that may occur in clinical practice, including over-estimation (with dilation), and under-estimation (with erosion).

**Figure 6:**
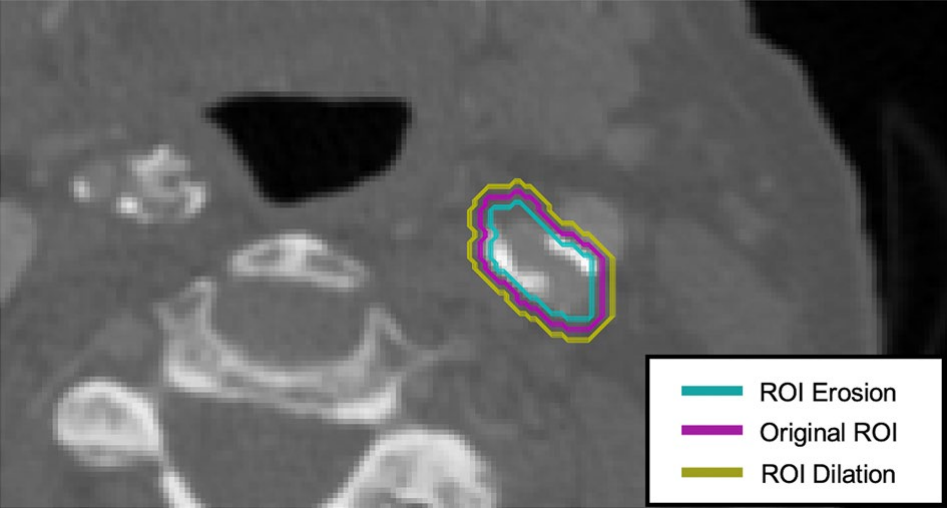
ROI segmentation and perturbations. Carotid CTA images were manually segmented to delineate the carotid artery. The original ROI was subjected to morphological operations: erosions and dilations aimed to assess the robustness of radiomic features to perturbations in image segmentation.

To achieve these perturbations, the original ROIs delineated by the primary reader (EPVL) were subjected to the dilation and erosion image morphological operations implemented in Python, see Fig. 6.

For single-slice analysis, we used a circular structuring element of radius 1, with iterations of 1–2 for ROI dilation and erosion. For multi-slice analysis, we used a spherical structuring element of radius 1, with iterations of 1–2 for ROI dilation, but only 1 iteration for ROI erosion in order to ensure that a sufficient number of pixels would be available for the downstream radiomic feature calculation after erosion. Where resegmentation was applied as a pre-processing scheme, ROI erosion was not performed, only ROI dilation to ensure that all ROIs had sufficient pixels for radiomic feature extraction, details of resegmentation are provided below.

#### Image pre-processing

Prior to radiomic feature calculations, there are different image pre-processing schemes that can be applied to a CTA scan. Three schemes were investigated: (a) Original image (no image pre-processing applied), (b) Normalisation and (c) Resegmentation.

Normalisation is generally a necessary image pre-processing step for magnetic resonance images since their grey values are arbitrary. In contrast, the grey values in CT images are already calibrated to HUs. However, CTA images may have differences in contrast filling and so we investigated the impact of prior image normalisation to the robustness of the extracted radiomic features. When investigating the image normalisation scheme, the CTA image was normalised such that the pixel values assumed an approximate Gaussian distribution.

Resegmentation refers to the process whereby only pixels within a specified grey value range are retained for radiomic feature calculation within the ROI/VOI^45^. Resegmentation was applied with an upper limit of 200, and a lower limit of 0 which restricted radiomic feature extraction to only the pixels with HU values between 0 and 200. This grey value range resegmentation aids with excluding the effects of excess carotid macro-calcification and limits the effect of luminal contrast and perivascular carotid fat within the CTA ROI/VOI. For resegmentation, we used a fixed BW of 25 only (PyRadiomics version 3.0 default) for image quantisation, described further below.

#### Image quantisation

Image quantisation refers to the conversion of image grey values to a discrete set of grey value counts. Before radiomic features are calculated, the image must be quantised by using a fixed number of bins, or by using a fixed BW. We varied the BWs of the image grey value histogram from 10 to 35, in increments of 5. For BN variations, we varied the fixed number of bins as follows: 8, 16, 32, 64, 128 and 256. This range of bin sizes was chosen based on the guidance in the PyRadiomics documentation^46,47^*.*

#### Multi-slice analysis: Image resampling and interpolation method

Higher-order radiomic feature extraction requires isotropic images, i.e. the pixel dimensions in the *x*, *y* and *z* directions are the same, to be rotationally invariant^48,49^. In CT imaging, images are often isotropic in-plane but will have a larger *z*-axis slice spacing and therefore be anisotropic in 3D. In radiomics studies, it is common for images to be isotropically resampled. We investigated the effect of using B-spline interpolation (PyRadiomics default) *versus* linear interpolation (faster and simpler) to resample the 3 mm slice thickness images and VOI segmentation masks to 1 × 1 × 1mm^3^ on the extracted radiomic features.

### Statistical analysis

For statistical comparisons between culprit *versus* non-culprit carotid arteries, the difference between the two paired groups were assessed for normality visually with histogram plots and statistically with the Shapiro–Wilk test. Where the normality assumption was met, the paired *t*-test was used, if not, the non-parametric Wilcoxon signed-rank test was used. A *p*-value < 0.05 was considered statistically significant.

The Dice coefficient (DC), a measure of segmentation overlap commonly used in computer vision and machine learning applications^50^, was calculated to assess agreement between ROI segmentations in the following ways: (1) comparing the ROIs for 8 carotid arteries drawn by the primary reader (EPVL) at two separate time points to determine intra-observer variability, (2) comparing ROIs for 8 carotid arteries drawn by the primary reader with those drawn by a second independent reader (CW) to determine inter-observer variability and (3) comparing the ROIs for 82 carotid arteries drawn by the primary reader with the ROIs generated following morphological operations (dilations and erosions) to determine the variability generated by systematic ROI perturbations.

The DC measures the level of agreement between different image segmentations by considering the level of overlap between ROI *X* and ROI *Y* over the total number of pixels in ROI *X* and ROI *Y* according to Eq. (1):

1 $$DC = \frac{{2\left| {X \cap Y} \right|}}{\left| X \right| + \left| Y \right|},$$

where $\left| \cdot \right|$ denotes the cardinality of the pixels contained in a certain set.

We measured the degree of robustness using the 2-way mixed-effects model, absolute agreement, single rater and the 2-way mixed-effects model, consistency, single rater intraclass correlation coefficient (ICC) according to the McGraw and Wong convention^51^ and according to the ICC guidelines of Koo and Li^52^, as appropriate.

Let *n* and *k* be the number of subjects and number of raters/measurements, respectively, the ICCs used are defined as follows:

- two-way mixed effects, consistency, single rater/measurement:
  2 $$ICC\left( {3,1} \right) = \frac{{MS_{R} - MS_{E} }}{{MS_{R} + \left( {k - 1} \right) MS_{E} }};$$
- two-way mixed effects, absolute agreement, single rater/measurement:
  3 $$ICC\left( {2,1} \right) = \frac{{MS_{R} - MS_{E} }}{{MS_{R} + \left( {k - 1} \right) MS_{E} + \frac{k}{n}\left( {MS_{C} - MS_{E} } \right)}};$$
  where $MS_{R}$, $MS_{E}$ and $MS_{C}$ are the mean square for rows, mean square for error and mean square for columns, respectively.

The ICC values fall between 0 and 1. Radiomic features were classified into three groups, with ICC values < 0.5, between 0.5 to 0.9, and ≥ 0.9, being indicative of poor, moderate and excellent robustness, respectively^53^.

All statistical analysis was performed in IBM SPSS Statistics for Macintosh and Python. Further details about the software and packages used are provided in Supplementary Methods S4.

#### Machine learning classification

Only the features with excellent robustness were used for the classification of culprit *versus* non-culprit carotid arteries. To reduce multicollinearity and feature redundancy, pairwise feature-to-feature correlations were determined using the Spearman Rank correlation. For pairs of features with a $Spearman | r_{s } | \ge 0.95,$ the feature with the highest AUC in univariate logistic regression was retained, and the latter was discarded^54^.

The features were subsequently standardised to have a mean of zero and a variance of one. 6 machine learning classifiers were evaluated, using a random state of 42 for reproducibility: decision tree^55^, random forest^56^, LASSO regression^57^, Elastic Net regression (weight for *L*_1_ and *L*_2_ penalties = 0.5)^58^, a neural network^59^ and XGBoost^60^. Further details about the machine learning classifier configurations are provided in Supplementary Methods S5. The dataset was shuffled and the average performance (accuracy and AUC) of the classifiers calculated following five-fold stratified cross-validation.

The AUC of the radiomics-only models, and of the integrated models (using radiomics features and calcium as predictors) were compared with the AUC of the calcium-only models using DeLong’s method^61^ to compare classifier performance for both single- and multi-slice approaches in each fold of the five-fold cross-validation scheme. The distribution of AUC values was compared using the Wilcoxon signed-rank test for the following comparisons: (1) calcium-only *versus* radiomics-only model, (2) calcium-only *versus* integrated model and (3) radiomics-only *versus* integrated model.

## Supplementary Information

Supplementary Information

## Data Availability

The anonymised datasets used in the current study are available from the corresponding author upon reasonable request.
