# Supplementary material for: Assessing robustness of carotid artery CT angiography radiomics in the identification of culprit lesions in cerebrovascular events
Source: Sci Rep. 2021 Feb 10;11:3499. doi: 10.1038/s41598-021-82760-w (PMC7876096; doi:10.1038/s41598-021-82760-w)
Supplement: Supplementary file 1 — Supplementary Information [file 41598_2021_82760_MOESM1_ESM.docx]

**Title**

Assessing Robustness of Carotid Artery CT Angiography Radiomics in the Identification of Culprit lesions in Cerebrovascular Events

**Full author list**

Elizabeth PV Le, Leonardo Rundo, Jason M Tarkin, Nicholas R Evans, Mohammed M Chowdhury, Patrick A Coughlin, Holly Pavey, Chris Wall, Fulvio Zaccagna, Ferdia A Gallagher, Yuan Huang, Rouchelle Sriranjan, Anthony Le, Jonathan R Weir-McCall, Michael Roberts, Fiona J Gilbert, Elizabeth A Warburton, Carola-Bibiane Schönlieb, Evis Sala, James HF Rudd

**Supplementary Material**

**Supplementary file contents:**

**Supplementary Table S1**

A breakdown of radiomic features by robustness category (excellent, moderate or poor), according to feature class type in single-slice analysis (original image and using a fixed bin width of 25 [PyRadiomics default setting]).

**Supplementary Table S2**

The 19 different image setting configurations investigated in single-slice analysis and multi-slice analysis.

**Supplementary Table S3**

Level of agreement (absolute agreement and consistency) between B-spline and linear interpolation by feature class in multi-slice analysis (original image and using a fixed bin width of 25 [PyRadiomics default setting]).

**Supplementary Table S4**

Radiomic features with excellent robustness to ROI/VOI segmentation perturbations and are non-redundant in different image settings were subsequently used for the binary classification of culprit *versus* non-culprit carotid arteries.

**Supplementary Table S5**

Distribution of carotid calcium across each fold in 5-fold cross-validation in training and test sets, stratified by culprit and non-culprit carotid arteries.

**Supplementary Table S6**

Mean sensitivity and specificity of the different machine learning classifiers in 5-fold cross-validation.

**Supplementary Table S7**

Radiomic features extracted from the ROI/VOIs in this study. All radiomic features were extracted using PyRadiomics[^32^](https://paperpile.com/c/CLgMSc/9xcHo) version 3.0 and the radiomic feature definitions and equations can be found on the online PyRadiomics documentation (<https://pyradiomics.readthedocs.io/en/latest/>).

**Supplementary Table S8**

Number and proportion of radiomic features extracted from each radiomic feature class.

**Supplementary Figure S1**

Violin plots of ICC(2,1) absolute agreement values for robustness against image segmentation perturbations in different image settings for single-slice and multi-slice analysis.

**Supplementary Figure S2**

Bar chart of single-slice analysis radiomic features that demonstrated excellent robustness across different image settings.

**Supplementary Figure S3**

Bar chart of multi-slice analysis radiomic features that demonstrated excellent robustness across different image settings.

**Supplementary Methods S1**

Details of culprit carotid identification and image acquisition protocol for contrast and non-contrast enhanced carotid CT images.

**Supplementary Methods S2**

Carotid artery plaque characteristics methodology, including calcium scoring, carotid stenosis measurement and carotid plaque type classification.

**Supplementary Methods S3**

Intra-and inter-observer reproducibility determination methodology.

**Supplementary Methods S4**

Details of software and Python packages used in this study for resampling, segmentation, robustness analysis and statistical analysis.

**Supplementary Methods S5**

Machine learning classifier configurations.

***Supplementary Table S1.*** *Break down of radiomic features by robustness category and feature class type in single-slice analysis*

| **ICC(2,1) absolute agreement** | **Number of radiomic features per feature class with:** | | |
| --- | --- | --- | --- |
| **Feature Class** | **Excellent Robustness**  **ICC** $\geq$**0.9** | **Moderate Robustness**  **0.5** $\leq$ **ICC < 0.9** | **Poor Robustness**  **ICC < 0.5** |
| First order (n=18) | 11 (61.1%) | 5 (27.8%) | 2 (11.1%) |
| GLCM (n=24) | 14 (58.3%) | 9 (37.5%) | 1 (4.2%) |
| GLDM (n=14) | 6 (42.9%) | 5 (35.7%) | 3 (21.4%) |
| GLRLM (n=16) | 9 (56.3%) | 4 (25.0%) | 3 (18.8%) |
| GLSZM (n=16) | 7 (43.8%) | 7 (43.8%) | 2 (12.5%) |
| NGTDM (n=5) | 2 (40.0%) | 3 (60.0%) | 0 (0.0%) |
| Total (n=93) | 49 (52.7%) | 33 (35.5%) | 11 (11.8%) |

*Image setting: original image, fixed bin width = 25; single-slice analysis*

***Supplementary Table S2.*** *19 different image setting configurations investigated in single-slice and multi-slice analysis*

| **#** | **Image Type** | **Image Quantisation Method** | **Quantisation Value** |
| --- | --- | --- | --- |
| 1 | Original | Bin Number | 8 |
| 2 | Original | Bin Number | 16 |
| 3 | Original | Bin Number | 32 |
| 4 | Original | Bin Number | 64 |
| 5 | Original | Bin Number | 128 |
| 6 | Original | Bin Number | 256 |
| 7 | Original | Bin Width | 10 |
| 8 | Original | Bin Width | 15 |
| 9 | Original | Bin Width | 20 |
| 10 | Original | Bin Width | 25 |
| 11 | Original | Bin Width | 30 |
| 12 | Original | Bin Width | 35 |
| 13 | Prior Normalisation | Bin Number | 8 |
| 14 | Prior Normalisation | Bin Number | 16 |
| 15 | Prior Normalisation | Bin Number | 32 |
| 16 | Prior Normalisation | Bin Number | 64 |
| 17 | Prior Normalisation | Bin Number | 128 |
| 18 | Prior Normalisation | Bin Number | 256 |
| 19 | Resegmentation | Bin Width | 25 |

*Original, refers to no prior image normalisation or resegmentation*

***Supplementary Table S3.*** *Level of agreement between B-spline and linear interpolation by feature class*

|  | **ICC(2,1) absolute agreement** | | | **ICC(3,1) consistency** | | |
| --- | --- | --- | --- | --- | --- | --- |
| **Feature Class** | **Excellent**  **ICC** $\geq$**0.9** | **Moderate**  **0.5 - 0.9** | **Poor**  **ICC <0.5** | **Excellent**  **ICC** $\geq$**0.9** | **Moderate**  **0.5 - 0.9** | **Poor**  **ICC <0.5** |
| First order (n=18) | 18 (100.0%) | 0 (0.0%) | 0 (0.0%) | 18 (100.0%) | 0 (0.0%) | 0 (0.0%) |
| GLCM (n=24) | 23 (95.8%) | 1 (4.2%) | 0 (0.0%) | 24 (100.0%) | 0 (0.0%) | 0 (0.0%) |
| GLDM (n=14) | 11 (78.6%) | 3 (21.4%) | 0 (0.0%) | 12 (85.7%) | 2 (14.3%) | 0 (0.0%) |
| GLRLM (n=16) | 12 (75.0%) | 4 (25.0%) | 0 (0.0%) | 13 (81.3%) | 3 (18.8%) | 0 (0.0%) |
| GLSZM (n=16) | 13 (81.3%) | 3 (18.8%) | 0 (0.0%) | 13 (81.3%) | 3 (18.8%) | 0 (0.0%) |
| NGTDM (n=5) | 5 (100.0%) | 0 (0.0%) | 0 (0.0%) | 5 (100.0%) | 0 (0.0%) | 0 (0.0%) |
| Total (n=93) | 82 (88.2%) | 11 (11.8%) | 0 (0.0%) | 85 (91.4%) | 8 (8.6%) | 0 (0.0%) |

*Image type: multi-slice analysis, original (no normalisation/resegmentation), fixed bin width 25*

***Supplementary Table S4.*** *Non-redundant radiomic features with excellent robustness in different image settings*

| **Multi-slice (B-spline interpolation, Bin width=25)** | |
| --- | --- |
| **Original (n=14)** | **Resegmentation (n=10)** |
| 1. First Order: 90th Percentile 2. First Order: Root Mean Squared 3. First Order: Maximum 4. First Order: Robust Mean Absolute Deviation 5. GLCM: Cluster Shade 6. GLCM: Cluster Tendency 7. GLCM: Inverse Variance 8. GLCM: Joint Average 9. GLDM: LargeDependenceEmphasis 10. GLDM: Large Dependence High Grey Level Emphasis 11. GLSZM: Small Area Emphasis 12. GLSZM: Size Zone NonUniformity 13. NGTDM: Busyness 14. NGTDM: Complexity | 1. First Order: Mean Absolute Deviation 2. GLCM: Difference Variance 3. GLCM: Joint Energy 4. GLDM: Dependence Variance 5. GLDM: Large Dependence High Grey Level Emphasis 6. GLRLM: Long Run High Grey Level Emphasis 7. GLRLM: Grey Level NonUniformity Normalised 8. GLSZM: Grey Level NonUniformity 9. GLSZM: Large Area High Grey Level Emphasis 10. GLSZM: Size Zone NonUniformity |
| **Single-slice (Bin width=25)** | |
| **Original (n=14)** | **Resegmentation (n=7)** |
| 1. First Order: Entropy 2. First Order: Range 3. First Order: Kurtosis 4. First Order: Skewness 5. First Order: Total Energy 6. GLCM: Joint Average 7. GLCM: Cluster Shade 8. GLCM: Inverse Variance 9. GLDM: Large Dependence High Grey Level Emphasis 10. GLRLM: Run Entropy 11. GLRLM: Long Run High Grey Level Emphasis 12. GLRLM: Run Percentage 13. GLSZM: Large Area High Grey Level Emphasis 14. GLSZM: Size Zone NonUniformity | 1. First Order: Energy 2. First Order: Total Energy 3. GLDM: Large Dependence High Grey Level Emphasis 4. GLRLM: Long Run High Grey Level Emphasis 5. GLSZM: Large Area Emphasis 6. GLSZM: Large Area High Grey Level Emphasis 7. GLSZM: Large Area Low Grey Level Emphasis |

***Supplementary Table S5.*** *Distribution of carotid calcium across each fold in 5-fold cross validation in training and test sets*

| **Train** | **Fold 1** | | **Fold 2** | | **Fold 3** | | **Fold 4** | | **Fold 5** | |
| --- | --- | --- | --- | --- | --- | --- | --- | --- | --- | --- |
|  | **CC** | **NC** | **CC** | **NC** | **CC** | **NC** | **CC** | **NC** | **CC** | **NC** |
| **Mean** | 434.4 | 448.8 | 431.2 | 451.4 | 411.6 | 394.3 | 445.2 | 418.2 | 417.0 | 400.0 |
| **SD** | 376.5 | 479.6 | 470.8 | 387.0 | 442.9 | 323.4 | 446.6 | 390.1 | 466.8 | 391.5 |
| **Min** | 0 | 0 | 0 | 0 | 0 | 0 | 0 | 0 | 1 | 0 |
| **Max** | 1480 | 1963 | 1963 | 1671 | 1963 | 1119 | 1963 | 1671 | 1963 | 1671 |
| **Test** | **Fold 1** | | **Fold 2** | | **Fold 3** | | **Fold** | | **Fold 5** | |
| **Mean** | 472.0 | 414.3 | 394.9 | 270.3 | 471.3 | 505.9 | 332.5 | 407.0 | 449.0 | 483.0 |
| **SD** | 676.9 | 366.1 | 333.0 | 300.4 | 457.6 | 561.8 | 431.6 | 330.4 | 335.2 | 313.4 |
| **Min** | 1 | 0 | 2 | 0 | 75 | 0 | 5 | 0 | 0 | 19 |
| **Max** | 1963 | 1119 | 884 | 768 | 1480 | 1671 | 1024 | 1087 | 877 | 877 |
| **AUC** | 0.37 | | 0.5 | | 0.55 | | 0.5 | | 0.25 | |

*SD, standard deviation; CC, culprit; NC, non-culprit*

***Supplementary Table S6.*** *Mean Sensitivity and Specificity of different machine learning classifiers in 5-fold cross-validation*

|  | 1. Calcium only | |
| --- | --- | --- |
| Classifier | Mean Sensitivity (%) | Mean Specificity (%) |
| Decision Tree | 38.9 | 48.9 |
| Random Forest | 46.1 | 48.9 |
| LASSO | 65.0 | 32.5 |
| ElasticNet | 42.5 | 50.0 |
| Neural Network | 43.9 | 61.4 |
| XGBoost | 50.8 | 46.7 |
|  | 1. Radiomics only | |
| Classifier | Mean Sensitivity (%) | Mean Specificity (%) |
| Decision Tree | 58.6 | 48.3 |
| Random Forest | 63.3 | 55.8 |
| LASSO | 55.8 | 52.8 |
| ElasticNet | 60.8 | 47.8 |
| Neural Network | 55.6 | 58.1 |
| XGBoost | 55.8 | 53.3 |
|  | 1. Radiomics and Calcium | |
| Classifier | Mean Sensitivity (%) | Mean Specificity (%) |
| Decision Tree | 60.8 | 43.6 |
| Random Forest | 53.3 | 63.3 |
| LASSO | 63.3 | 55.3 |
| ElasticNet | 63.3 | 55.3 |
| Neural Network | 58.1 | 53.1 |
| XGBoost | 53.3 | 55.6 |

*Image setting: multi-slice analysis with resegmentation and a fixed bin width of 25.*

***Supplementary Table S7.*** *Radiomic features extracted (n=93)*

| **#** | **Radiomic Feature** | **#** | **Radiomic Feature** | **#** | **Radiomic Feature** |
| --- | --- | --- | --- | --- | --- |
| ***First-Order*** | | 32 | IMC 1: Information Measures of Correlation | 63 | Long Run Low Grey Level Emphasis |
| 1 | 10th Percentile | 33 | IMC 2: Information Measures of Correlation | 64 | Low Grey Level Run Emphasis |
| 2 | 90th Percentile | 34 | Inverse Variance | 65 | Run Entropy |
| 3 | Energy | 35 | Joint Average | 66 | Run Length NonUniformity |
| 4 | Entropy | 36 | Joint Energy | 67 | Run Length NonUniformity Normalised |
| 5 | Interquartile Range | 37 | Joint Entropy | 68 | Run Percentage |
| 6 | Kurtosis | 38 | MCC | 69 | Run Variance |
| 7 | Maximum | 39 | Maximum Probability | 70 | Short Run Emphasis |
| 8 | Mean Absolute Deviation | 40 | Sum Average | 71 | Short Run High Grey Level Emphasis |
| 9 | Mean | 41 | Sum Entropy | 72 | Short Run Low Grey Level Emphasis |
| 10 | Median | 42 | Sum Squares | ***GLSZM*** | |
| 11 | Minimum | ***GLDM*** | | 73 | Grey Level NonUniformity |
| 12 | Range | 43 | Dependence Entropy | 74 | Grey Level NonUniformity Normalised |
| 13 | Robust Mean Absolute Deviation | 44 | Dependence NonUniformity | 75 | Grey Level Variance |
| 14 | Root Mean Squared | 45 | Dependence NonUniformity Normalised | 76 | High Grey Level Zone Emphasis |
| 15 | Skewness | 46 | Dependence Variance | 77 | Large Area Emphasis |
| 16 | Total Energy | 47 | Grey Level NonUniformity | 78 | Large Area High Grey Level Emphasis |
| 17 | Uniformity | 48 | Grey Level Variance | 79 | Large Area Low Grey Level Emphasis |
| 18 | Variance | 49 | High Grey Level Emphasis | 80 | Low Grey Level Zone Emphasis |
| ***GLCM*** | | 50 | Large Dependence Emphasis | 81 | Size Zone NonUniformity |
| 19 | Autocorrelation | 51 | Large Dependence High Grey Level Emphasis | 82 | Size Zone NonUniformity Normalised |
| 20 | Cluster Prominence | 52 | Large Dependence Low Grey Level Emphasis | 83 | Small Area Emphasis |
| 21 | Cluster Shade | 53 | Low Grey Level Emphasis | 84 | Small Area High Grey Level Emphasis |
| 22 | Cluster Tendency | 54 | Small Dependence Emphasis | 85 | Small Area Low Grey Level Emphasis |
| 23 | Contrast | 55 | Small Dependence High Grey Level Emphasis | 86 | Zone Entropy |
| 24 | Correlation | 56 | Small Dependence Low Grey Level Emphasis | 87 | Zone Percentage |
| 25 | Difference Average | ***GLRLM*** | | 88 | Zone Variance |
| 26 | Difference Entropy | 57 | Grey Level NonUniformity | ***NGTDM*** | |
| 27 | Difference Variance | 58 | Grey Level NonUniformity Normalised | 89 | Busyness |
| 28 | ID | 59 | Grey Level Variance | 90 | Coarseness |
| 29 | IDM | 60 | High Grey Level Run Emphasis | 91 | Complexity |
| 30 | IDMN: Inverse Difference Moment Normalised | 61 | Long Run Emphasis | 92 | Contrast |
| 31 | IDN: Inverse Difference Normalised | 62 | Long Run High Grey Level Emphasis | 93 | Strength |

*GLCM, grey level co-occurrence matrix; GLDM, grey level dependence matrix; GLRLM, grey level run length matrix; GLSZM, grey level size zone matrix; NGTDM, neighbouring grey-tone difference matrix*

***Supplementary Table S8.*** *Number and proportion. of radiomic features extracted from each radiomic feature class*

| **Radiomic Feature Class** | **Number of Features Extracted** |
| --- | --- |
| ***First-order*** | 18 (19.4%) |
| ***GLCM*** | 24 (25.8%) |
| ***GLDM*** | 14 (15.1%) |
| ***GLRLM*** | 16 (17.2%) |
| ***GLSZM*** | 16 (17.2%) |
| ***NGTDM*** | 5 (5.4%) |
| **Total** | 93 (100.0%) |


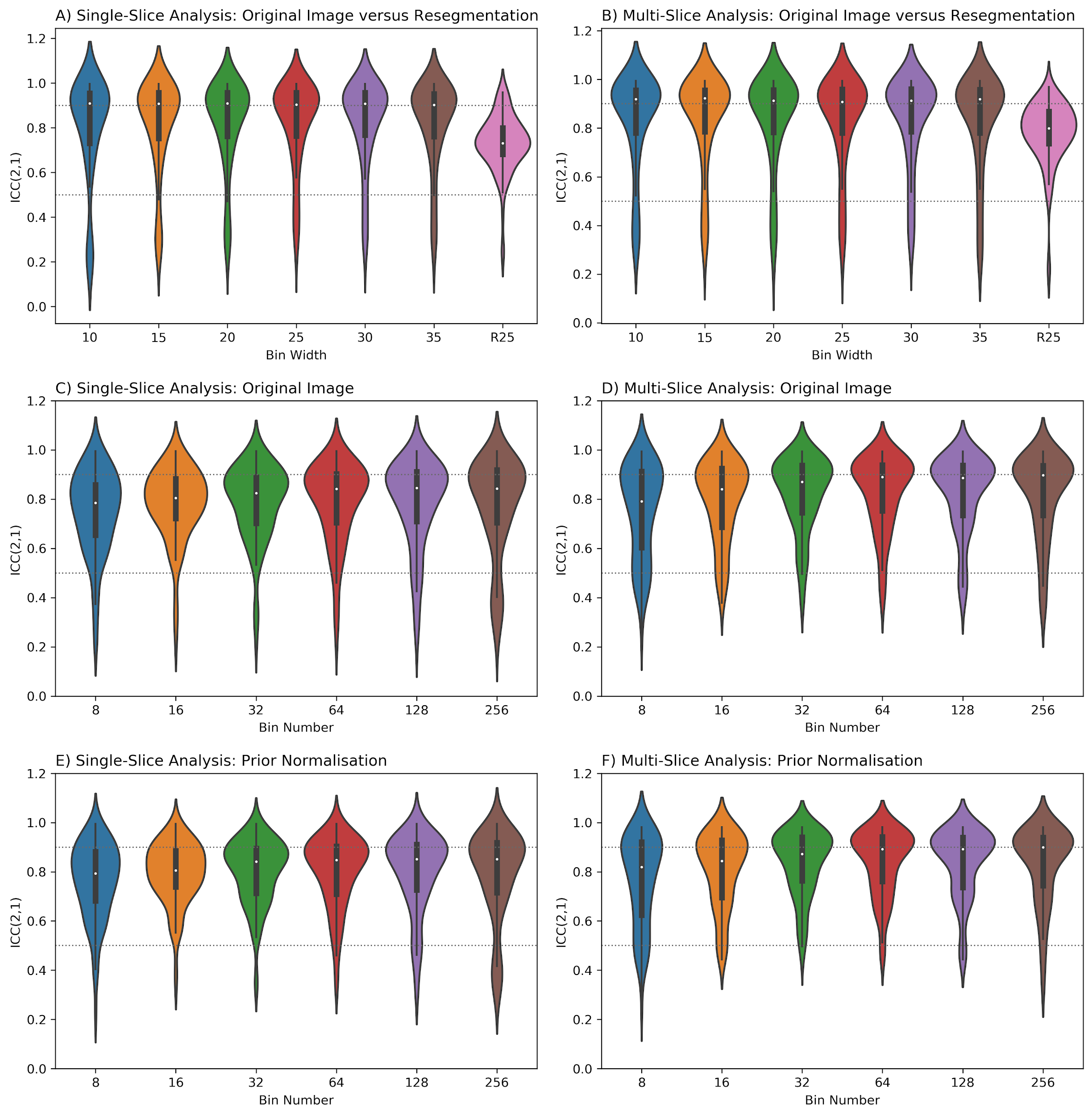
***Supplementary Figure S1. Violin plots of ICC(2,1) absolute agreement for robustness against ROI perturbations in different settings for single-slice and multi-slice analysis.*** *The median ICC(2,1) value is shown as a white dot. Original image refers to no normalisation of the image before radiomic feature extraction. Resegmentation refers to confining the radiomic feature extraction to a set of values between [0, 1, …, 200] HU only in the ROI/VOI. R25 refers to prior resegmentation and using a fixed bin width of 25 (PyRadiomics default). The horizontal line at ICC(2,1)=0.5 denotes the threshold between radiomic features with poor robustness and moderate robustness against ROI perturbations (ROI dilations and ROI erosions), the horizontal line at ICC(2,1)=0.9 denotes the threshold for a radiomic feature with excellent robustness.*

**
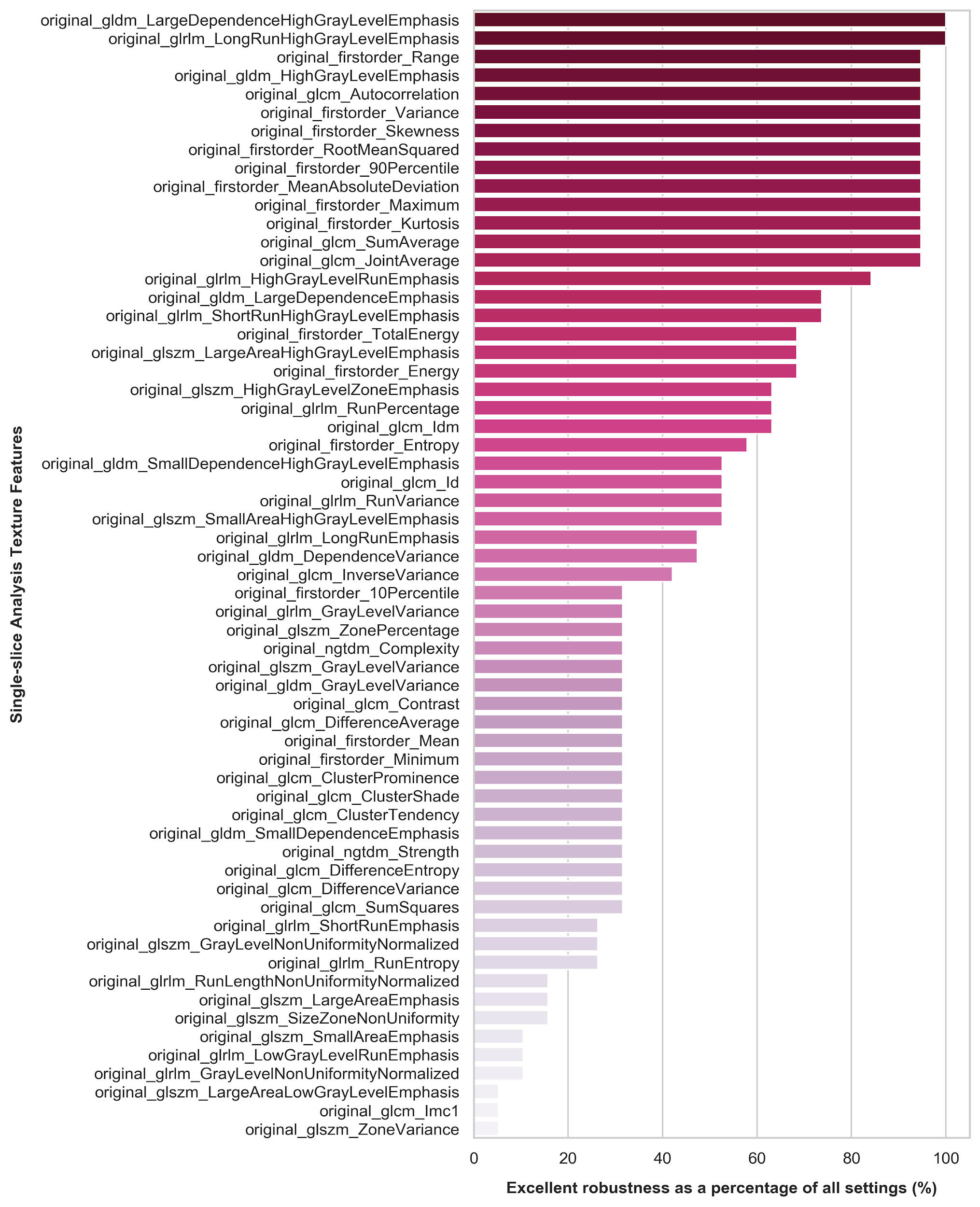
**

***Supplementary Figure S2. Bar chart of single-slice analysis radiomic features that demonstrated excellent robustness against ROI perturbations across different settings.*** *Only 2 radiomic features demonstrated excellent robustness across all 19 settings (100%). 61 out of 93 extracted radiomic features demonstrated excellent robustness in at least 1 setting.*

**
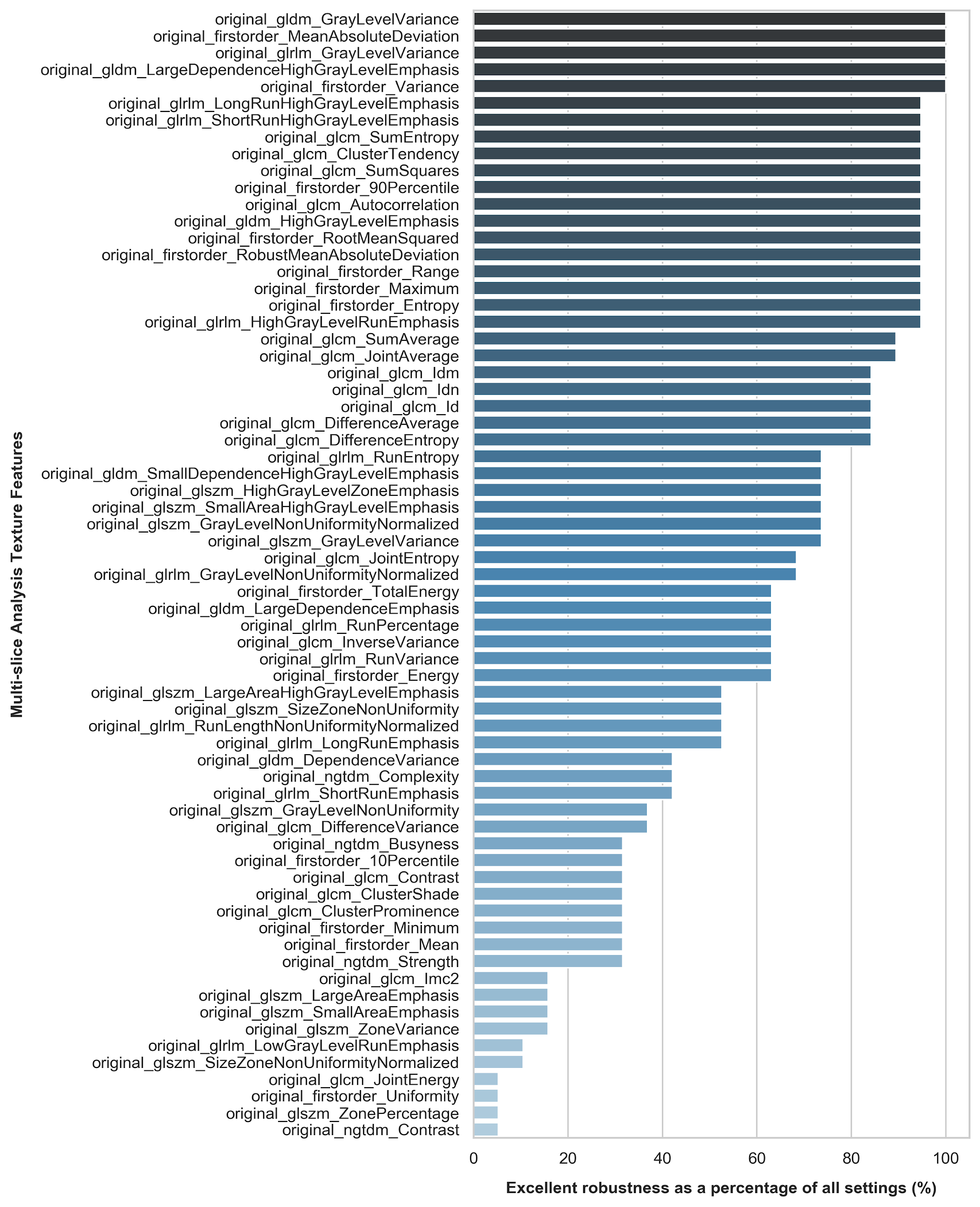
**

***Supplementary Figure S3. Bar chart of multi-slice analysis radiomic features that demonstrated excellent robustness against ROI perturbations across different settings.*** *Excellent robustness was defined as ICC(2,1)*$\geq$*0.9 comparing the original ROI with ROI dilation (1 and 2 iterations) and ROI erosion (1 iteration only).*

***Supplementary Methods S1. Culprit Carotid Identification and Image Acquisition Protocols***

Culprit carotid arteries were identified in patients with recent ischaemic TIA/stroke by the attending clinical team, including the stroke physician, neuroradiologist and/or neurovascular surgeon, taking into consideration all available clinical data including the side of neurological deficit at time of clinical presentation, carotid artery and brain imaging, echocardiography to exclude intra-cardiac thrombus and cardiac monitoring to exclude atrial fibrillation. As such, the clinically adjudicated diagnosis was the gold-standard for defining carotid ischaemic stroke and for identifying the culprit artery. This clinical diagnosis was made independently of the study investigators. In addition, all stroke patients had MRI-confirmed strokes in a distribution consistent with artery-to-artery embolization (i.e. lacunar infarcts or bilateral distributions suggesting a cardioembolic source were excluded). Furthermore, individuals with stroke had prolonged cardiac monitoring in both the inpatient setting and during subsequent investigations. Individuals with atrial fibrillation detected subsequently were excluded from analysis.

All imaging was performed on a GE Discovery combined 690 PET-CT combined scanner with an integrated 64-slice CT scanner (GE Healthcare, Waukesha, WI, USA). CTA images were acquired from the aortic arch to the Circle of Willis, using bolus tracking (triggered at 100 HU above baseline) with a region-of-interest placed in the aortic arch and 70-100 ml NIOPAM 300 (Bracco UK Limited, High Wycombe, UK) radiocontrast injected at 5 ml/second, followed by a 50 ml chaser of normal saline. Acquisition parameters: tube voltage 120 kV, maximum tube current 200 mA, rotation time of 0.8 second, pitch 0.969:1, in-plane pixel spacing of minimum 0.30×0.30 mm^2^ to maximum 0.59×0.59 mm^2^, slice thickness 0.625 mm with reconstruction interval (i.e. spacing between slices) of 0.4 mm.

Unenhanced CT images were acquired from the arch of the aorta to the external auditory meatus. CT acquisition parameters: tube voltage 120 kV, tube current 40 mA, rotation time 0.5 seconds, pitch 1.375, direct field of view 50 cm, section thickness 3.75 mm reconstructed to 3.27 mm.

***Supplementary Methods S2. Carotid Artery Plaque Characteristics***

Carotid calcium measured with the ‘Calcium Scoring’ plug-in of OsiriX on unenhanced CT images[^26^](https://paperpile.com/c/CLgMSc/4Tf3Y) acquired in the same setting as CTA Image Acquisition. The plug-in quantifies vascular macro-calcification according to the Agatston method,[^57^](https://paperpile.com/c/CLgMSc/coIo) which is commonly used for coronary artery calcification quantification and measured in Agatston units (AU). The calcification detection threshold is based on a CT attenuation value of 130 HU.

The degree of carotid artery stenosis as a percentage, was calculated according to the NASCET criteria[^58^](https://paperpile.com/c/CLgMSc/YsVZ) on CTA images (Equation S1):

| $\frac{B-A}{A}\times100\%$, | (S1) |
| --- | --- |

where *A* is the minimum luminal diameter at the point of maximal stenosis, and *B* is the diameter of the normal distal internal carotid artery.

Carotid lesions were classified according to established coronary CT criteria for plaque composition (calcified, non-calcified or mixed plaque)[^59^](https://paperpile.com/c/CLgMSc/ZItR).

***Supplementary Methods S3. Intra-and Inter-observer reproducibility***

The primary reader (EPVL) manually segmented 10% of carotid arteries (n = 8) that were randomly selected to determine intra-observer variability in ROI delineation. In addition, a second reader (Dr Chris Wall, Academic Cardiology Registrar, University of Cambridge), also manually segmented a random selection of carotid arteries (n = 8). This intra- and inter-observer variability in ROI delineation was used to ensure that the variations achieved using morphological operations (ROI erosion and dilation) reflected the variability that occurred in practice with human operators.

***Supplementary Methods S4. Software and Python details***

For multi-slice analysis, the initial resampling of CTA slices to 3 mm slice thickness used the OsiriX MD software resampling plugin (Version 10.0.3., Pixmeo SARL, Bernex, Geneva, Switzerland). Manual segmentations of the carotid arteries were conducted using TexRad (Feedback Medical Ltd, Cambridge, UK). Radiomic features were extracted using PyRadiomics version 3.0.

For robustness analysis, the morphological operations applied to the ROIs were implemented in Python 3.7.4 using the Python package, SciPy morphology and its ‘binary_dilation’ and ‘binary_erosion’ functions. Where normalisation was applied, the image was normalised by using the PyRadiomics ‘normalizeImage’ function, which leads to the pixel values assuming an approximate Gaussian distribution. This function sets the image pixel values to a mean of zero and a variance of one (i.e. z-score normalisation), by subtracting the mean of all grey values in the image from each pixel value and dividing by the standard deviation of those grey values.

All statistical analysis was performed in IBM SPSS Statistics for Macintosh (Version 25.0 Armonk, NY, USA: IBM Corp.) and Python 3.7.4.

***Supplementary Methods S5. Machine learning classifier configurations***

The following classifiers were implemented using the default configurations in scikit-learn version 0.23.1, which are documented in <https://scikit-learn.org/stable/tutorial/index.html>, with a random state of 42: decision tree (criterion: Gini Impurity), random forest (criterion: Gini Impurity) and neural network (1 hidden layer with 100 hidden units using the ReLU activation function, initial learning rate = 0.001, Adam optimizer and maximum number of iterations = 200). For LASSO regression, the norm used in penalisation was set to ‘L1’ for the sklearn default logistic regression classifier, whilst it was set to ‘elasticnet’ with an L1 ratio of 0.5 for Elastic Net regression. The SAGA solver was used for the logistic regression-based classifiers. The XGBoost classifier was implemented using the default settings provided in the XGBoost Python package version 1.2.0, detailed in <https://xgboost.readthedocs.io/en/latest/index.html>.
